# Lung cancer risk and pollution in an industrial region of Northern Spain: a hospital-based case-control study

**DOI:** 10.1186/1476-072X-10-10

**Published:** 2011-01-25

**Authors:** María Felicitas López-Cima, Javier García-Pérez, Beatriz Pérez-Gómez, Nuria Aragonés, Gonzalo López-Abente, Adonina Tardón, Marina Pollán

**Affiliations:** 1 Cancer and Environmental Epidemiology Unit, National Center for Epidemiology, Carlos III Institute of Health, Avda. Monforte de Lemos, 5, 28029 Madrid, Spain; 2 CIBER en Epidemiología y Salud Pública (CIBERESP), Spain; 3 Molecular Epidemiology of Cancer Unit, University Institute of Oncology, University of Oviedo, C/Fernando Bongera, s/n, 33006 Oviedo, Spain

## Abstract

**Background:**

Asturias, an Autonomous Region in Northern Spain with a large industrial area, registers high lung cancer incidence and mortality. While this excess risk of lung cancer might be partially attributable to smoking habit and occupational exposure, the role of industrial and urban pollution also needs to be assessed. The objective was to ascertain the possible effect of air pollution, both urban and industrial, on lung cancer risk in Asturias.

**Methods:**

This was a hospital-based case-control study covering 626 lung cancer patients and 626 controls recruited in Asturias and matched by ethnicity, hospital, age, and sex. Distances from the respective participants' residential locations to industrial facilities and city centers were computed. Using logistic regression, odds ratios (ORs) and 95% confidence intervals (95%CIs) for categories of distance to urban and industrial pollution sources were calculated, with adjustment for sex, age, hospital area, tobacco consumption, family history of cancer, and occupation.

**Results:**

Whereas individuals living near industries displayed an excess risk of lung cancer (OR = 1.49; 95%CI = 0.93-2.39), which attained statistical significance for small cell carcinomas (OR = 2.23; 95%CI = 1.01-4.92), residents in urban areas showed a statistically significant increased risk for adenocarcinoma (OR = 1.92; 95%CI = 1.09-3.38). In the Gijon health area, residents in the urban area registered a statistically significant increased risk of lung cancer (OR = 2.17; 95%CI = 1.25-3.76), whereas in the Aviles health area, no differences in risk were found by area of exposure.

**Conclusions:**

This study provides further evidence that air pollution is a moderate risk factor for lung cancer.

## Background

Lung cancer is the most common cause of cancer-related death. In Spain, it accounted for almost 20,000 deaths in 2007, amounting to 27% of all cancer deaths in males and 7% in females [1]. This cancer displays marked geographic variability, and Asturias, located in Northern Spain, is one of the regions registering clear excess mortality [2], with adjusted mortality rates ranking among the highest in Spain for both sexes [3]. This is a heavily industrialized area, with metal industries, coal mining facilities, and fossil-fuel-fired power plants.

While tobacco use is the main risk factor for lung cancer, and available estimates attribute 80% to 90% of cases in men and 55% to 80% of cases in women to cigarette smoking [4], there are other well-known lung carcinogens, such as radon [5,6], arsenic, asbestos, heavy metals -chromium VI, nickel, cadmium- coke oven and coal gasification fumes and soot [7]. Accordingly, occupational exposures in industrial facilities have been held to account for a further 9% to 15% of cases [8].

Furthermore, industries may pose a risk, not only to workers, but also to persons residing in their proximity, since their emissions, which release toxic substances to the environment, are an important source of air pollution [9-12]. Several studies have reported an association between risk of lung cancer and proximity to certain industries, such as iron and steel foundries or chemical, petrochemical and coke oven plants [13-16]. In this connection, a recent study reported increased lung cancer mortality in the proximity of a combustion installation located in Asturias [17].

Urban air pollution, particularly due to traffic- and heating-related emissions in these areas, has also come to be viewed as a risk factor for developing lung cancer [18-20]. Three cohort studies conducted in the USA during the 1990s reported a link between several air pollution indicators and cancer risk among urban residents [21-23]. In Europe, a case-control study conducted in an industrialized town in Northern Italy showed an increased risk for lung cancer among city residents living in the most polluted areas versus those living in less polluted areas [24].

Due to the high occurrence of lung cancer in Asturias, CAPUA (Cáncer de Pulmón en Asturias - Lung Cancer in Asturias), a hospital-based case-control study, was set in motion to furnish in-depth knowledge of the causes of this excess. In this paper, we examine the effects of air pollution, urban and industrial, on lung cancer risk in Asturias.

## Methods

### Study subjects

The CAPUA study is a hospital-based case-control study conducted at the Oviedo University's Molecular Epidemiology of Cancer Unit, University Institute of Oncology. Details of the study design and methods have been described elsewhere [25-27]. Briefly, patients were recruited at four public hospitals in Asturias, each of which is the reference center for the surrounding catchment health area (i.e., the respective administrative health division). The four hospitals were: the Cabueñes Hospital in the city of Gijon (262,470 inhabitants); the San Agustin Hospital in the town of Aviles (78,989 inhabitants); the General Hospital in the city of Oviedo (187,093 inhabitants); and the Alvarez-Buylla Hospital in the town of Mieres (24,956 inhabitants) [28]. Each hospital attends to the residents of its designated catchment area, which includes the relevant host town or city plus all smaller outlying municipalities coming within the geographical boundaries defined by the health authorities. From October 2000 to October 2008, a standard protocol was used to recruit a total of 878 incident cases of histologically confirmed lung cancer, along with 672 controls individually matched to the cases by ethnicity, hospital, sex, and age (±5 years). Controls were selected among patients admitted to hospitals for acute health conditions unrelated to the exposures of interest. The most frequent diseases or conditions of the controls were as follows: 38.2% inguinal or abdominal hernias (International Classification of Diseases-9th revision (ICD-9): 550-553); 33.4% injuries (ICD-9: 800-848, 860-869, 880-897) - mainly fractures (90.2%), fundamentally due to accidental falls (in particular, 22.4% pelvis fractures, 10.4% arm fractures and 42.6% leg fractures) -; and 13.2% intestinal obstructions (ICD-9: 560, 569, 574). Both cases and controls were required to reside within the recruiting hospital's assigned geographic health area. The study was approved by the ethics committees of the various hospitals, and written consent was obtained from all participants.

### Data-collection

Information on known or potential risk factors for lung cancer was collected personally through computer-assisted questionnaires by trained interviewers during patients' first hospital admission for diagnosis. Structured questionnaires collected data from each participant on age, sex, sociodemographic characteristics, residential history (including address of last residence), current and past tobacco use, personal and family history of cancer, and occupational history.

Participants were categorized by tobacco consumption into three groups, namely: never smokers, defined as subjects who had not smoked at least one cigarette per day regularly for six months or longer in their lifetimes; former smokers, defined as regular smokers who had stopped smoking at least five years before the interview; and current smokers defined as subjects who met none of these criteria. Smoking intensity (pack-years (PY)) was defined as the number of packs of cigarettes smoked per day multiplied by the number of years of smoking. Subjects were also categorized as light (<38 PY) or heavy (≥38 PY) smokers, based on mean cumulative tobacco consumption in the control group. Finally, smoking status and intensity were combined into a joint variable having the following five levels: never smokers; former smokers <38 PY; current smokers <38 PY; former smokers ≥38 PY; and current smokers ≥38 PY.

For each job held for a minimum of 6 months or longer, we obtained information on industry name, production type, job title, and the year in which the job began and ended. Occupations and industries were coded using the 1977 Standard Occupational Classification [29] and 1972 Standard Industrial Classification schemes [30]. Lastly, each coded occupation was categorized as a high-risk or non high-risk occupation for lung cancer in accordance with published literature. Thus, to study occupational history, participants were classified into three groups, namely, unexposed individuals that had never worked in a high-risk job, subjects that had worked <35 years in a high-risk job, and finally, subjects that had worked ≥35 years in a high-risk job. This cut-off was based on the 75th percentile of time spent in high-risk jobs by the control group.

### Geographic analysis

Each participant's last residence was geocoded using BatchGeo [31] and the Spanish Farm Plot Geographic Information System (Sistema de Información Geográfica de Parcelas Agrícolas - SIGPAC) [32]. To measure distances, a geodesic calculator was used to convert BatchGeo WGS84-projection coordinates (longitude/latitude) into the Universal Transverse Mercator (UTM) Zone 30 (ED50) coordinates used by SIGPAC.

Of the 1550 participants interviewed (878 cases and 672 controls), 1481 individuals (839 cases and 642 controls) were geocoded, 1249 using BatchGeo and 232 using SIGPAC. A total of 213 cases without matched controls and 16 controls without matched cases were excluded from the analyses (these persons had similar characteristics that those included in the study). Thus, the final study population available for study comprised 626 matched pairs, all the members of which were Caucasian.

Data on industries were obtained from the European Pollutant Emission Register (EPER) [33,34]. This is a public inventory of industries set up by the European Commission under the terms of Directive 96/61/EC, which provides information about the location and emissions of industrial pollution of all industrial plants that have exceeded the reporting thresholds for one or more of the pollutants included in EU Decision 2000/479/CE. In a previous study, our team validated and corrected the geographical coordinate data provided by the EPER for all Spanish industries [35]. We identified a total of 23 industrial installations that had reported releases to air in 2001 in the health areas targeted, though, due to the specific characteristics of one of these industries, the installation in question was divided into 4 different sections spread over 7 kilometers. As a result, 26 industrial locations were included in the analysis. Data on the date of commencement of industrial activity were obtained from the official websites of the industrial companies themselves.

For each subject, the following Euclidean distances were calculated: a) urban nucleus distance, i.e., the distance between the subject's last residence and the centroid of the town in which the hospital was situated; and b) industrial distance, i.e., the shortest distance between the subject's residence and any of the previously mentioned 26 industrial installations.

The distribution of these distances among controls was used to define the boundaries of the geographic areas of interest, in line with the methodology proposed by Barbone [24]:

1) the industrial area being the area defined by the first decile of industrial distance;

2) the urban area being the area defined by the first decile of urban distance;

3) the semi-urban area being the area defined by the second decile of urban distance; and,

4) the reference area, being those zones not included above and corresponding mainly to rural settings.

Participants were thus deemed to be exposed to industrial, urban or semi-urban pollution if their residence lay within one of these areas, which did not overlap.

### Data analysis

Multiple unconditional logistic regression models were used to estimate odds ratios (ORs) and 95% confidence intervals (95%CIs), in order to evaluate the possible relationship between lung cancer and urban and industrial distances, duly adjusting for matching factors (age, sex, and hospital area) and other potential confounding variables, such as smoking, occupation, and family history of cancer (classified into three levels, i.e., none, first-degree relatives with other types of cancer, and first-degree relatives with lung cancer). As we have considered a frequency matched study, given that matching conditions are very general and controls can fit the criteria for more than one case (the corresponding pairs can be interchangeable), the standard methodology is to use unconditional logistic regression including in the model the matched characteristics.

## Results

The analysis covered 626 lung cancer cases and 626 controls drawn from the Caucasian population of Asturias. Distribution by sex, age, hospital area, smoking history (smoking status, smoking intensity, and tobacco consumption), family history of cancer, occupational history, and histologic type of case is summarized in Table 1. There were more current smokers (68.7% vs. 48.9%) and more heavy smokers (62.01 vs. 38.27 PY) among cases than among controls (P < 0.001). Histologically, squamous cell carcinoma (39.4%) and adenocarcinoma (29.6%) were the main types of lung cancer.

**Table 1:** Characteristics of lung cancer cases and controls

Characteristic	Cases (n = 626) n (%)	Controls (n = 626) n (%)	*P*^a^
Sex			
Male	541 (86.4)	541 (86.4)	
Female	85 (13.6)	85 (13.6)	1.000
Age (yrs), mean (SD)	64.48 (10.96)	63.61 (11.21)	0.161
median (IQR)	65.00 (17.00)	64.00 (18.00)	0.172
Hospital area^b^			
Gijon (Cabueñes Hospital)	355 (56.7)	355 (56.7)	
Aviles (San Agustin Hospital)	176 (28.1)	176 (28.1)	
Oviedo (General Hospital)	58 (9.3)	58 (9.3)	
Mieres (Alvarez-Buylla Hospital)	37 (5.9)	37 (5.9)	1.000
Smoking Status			
Never	47 (7.5)	182 (29.1)	
Ever	579 (92.5)	444 (70.9)	<0.001
Former	181 (31.3)	227 (51.1)	
Current	398 (68.7)	217 (48.9)	<0.001
PY^c^, mean (SD)	62.01 (35.34)	38.27 (31.96)	<0.001
median (IQR)	55.00 (40.75)	32.00 (35.86)	<0.001
Smoking			
Never	47 (7.6)	182 (29.6)	
Former < 38 PY	62 (10.0)	151 (24.6)	
Current < 38 PY	72 (11.7)	102 (16.6)	
Former ≥ 38 PY	117 (18.9)	70 (11.4)	
Current ≥ 38 PY	320 (51.8)	109 (17.8)	<0.001
Family history of cancer			
None	338 (56.2)	371 (59.8)	
Other cancers	190 (31.6)	204 (32.9)	
Lung cancer	73 (12.2)	45 (7.3)	0.015
Histologic type			
Squamous cell carcinoma	242 (39.4)		
Adenocarcinoma	182 (29.6)		
Small cell carcinoma	109 (17.7)		
Large cell carcinoma	18 (2.9)		
Non-differentiated	42 (6.8)		
Others	14 (2.3)		
Clinical diagnosis	8 (1.3)		
Missing	11		
Worker in high-risk occupation			
Never	220 (35.4)	245 (39.3)	
Ever	401 (64.6)	379 (60.7)	0.162
Time in high-risk occupation^d^, mean (SD)	26.31 (14.59)	24.24 (13.95)	0.045
Median (IQR)	29.00 (25.00)	26.00 (23.00)	0.036
Population living in their last-residence			
for more than 5 years	547 (88.90)	552 (89.30)	0.903
for more than 10 years	492 (80.00)	495 (80.10)	0.977

^a^Two-sided χ^2 ^test, and Mann-Whitney test where appropriate

^b^Hospital area refers to each hospital's health catchment area

^c^Pack-years for ever-smokers

^d^Time (in years) for ever-workers

Table 2 shows the association between smoking, family history of cancer, occupational history and lung cancer broken down by histologic type. While tobacco consumption was strongly associated with all histologic types, the association was even stronger with squamous cell carcinoma. With regard to family history of lung cancer, this was associated with all types of lung cancer but the OR was statistically significant only for squamous cell carcinoma, probably due to the higher number of cases. Lastly, occupational history proved to be a risk factor for squamous cell carcinoma and adenocarcinoma, though, like family history above, it too was statistically significant solely in the case of squamous cell carcinoma.

**Table 2:** Odds ratios of lung cancer by histologic type and distribution of potential confounding variables

	Total				Squamous cell carcinoma			Adenocarcinoma			Small cell carcinoma		
	**Controls**	**Cases**	**OR (95%CI)**^**a**^	**P-value**	**Cases**	**OR (95%CI)**^**a**^	**P-value**	**Cases**	**OR (95%CI)**^**a**^	**P-value**	**Cases**	**OR (95%CI)**^**a**^	**P-value**
	**N**	**N**			**N**			**N**			**N**		

**Smoking**													
**Never**	182	47	Reference		7	Reference		21	Reference		6	Reference	
**Former < 38 PY**	151	62	2.84 (1.71-4.74)		22	7.63 (2.75-21.16)		14	1.63 (0.78-3.40)		6	2.34 (0.77-7.09)	
**Current < 38 PY**	70	72	5.29 (3.14-8.91)		23	11.65 (4.10-33.06)		24	3.71 (1.85-7.44)		7	5.46 (1.91-15.57)	
**Former** ≥ **38 PY**	102	117	13.00 (7.63-22.15)		53	36.63 (13.14-102.13)		28	7.64 (3.65-16.03)		16	12.88 (4.38-37.87)	
**Current** ≥ **38 PY**	109	320	23.74 (14.48-38.94)	<0.001^b^	111	57.78 (21.46-155.58)	<0.001^b^	69	12.12 (6.25-23.52)	<0.001^b^	54	28.15 (10.65-74.39)	<0.001^b^

**Family history of cancer**													
**None**	371	338	Reference		108	Reference		90	Reference		57	Reference	
**Other cancers**	204	190	1.02 (0.77-1.36)	0.607	77	1.46 (0.99-2.15)	0.161	48	0.86 (0.57-1.30)	0.885	21	0.67 (0.39-1.16)	0.319
**Lung cancer**	45	73	1.59 (1.00-2.51)	0.083	25	2.52 (1.38-4.61)	0.035	16	1.38 (0.72-2.66)	0.080	9	1.33 (0.59-3.01)	0.430

**Occupation**													
**Never**	245	220	Reference		68	Reference		76	Reference		47	Reference	
**< 35 years**	274	257	1.09 (0.79-1.49)		112	1.36 (0.88-2.10)		61	0.97 (0.61-1.54)		42	0.76 (0.44-1.31)	
≥ **35 years**	102	140	1.42 (0.96-2.10)	0.082^b^	60	1.74 (1.04-2.92)	0.048^b^	43	1.63 (0.94-2.82)	0.089^b^	17	0.75 (0.37-1.53)	0.384^b^

^a^ORs were estimated from a multiple logistic regression model that included age, sex, hospital area, tobacco consumption, family history of cancer, occupation, and exposure area

^b^Test for trend

**Figure 1:**
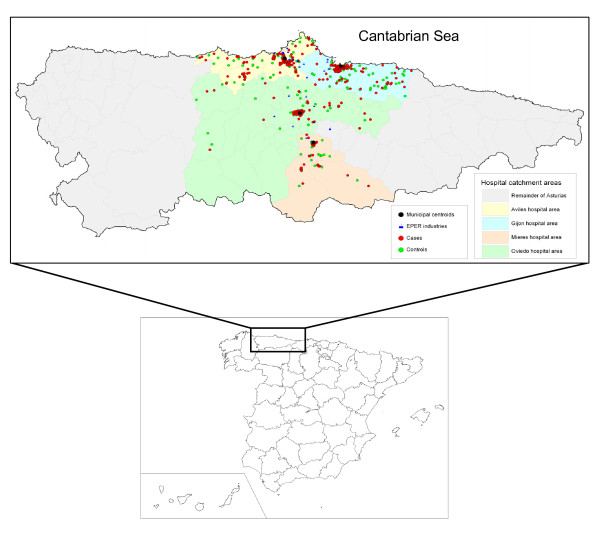
**Geographic distribution of cases, controls, industrial installations, and centroids in the four health areas**.

Locations of industrial installations, town centroids of the hospitals targeted, and residences of cases and controls are depicted in Figure 1.

Estimated ORs of lung cancer, both overall and by histologic subtype, are shown in Table 3 by pollution exposure category. Individuals living near industrial installations registered a non-statistically significant excess risk of lung cancer (adjusted-OR = 1.49; 95%CI = 0.93-2.39), a finding that mainly reflects the high risk observed for small cell carcinoma (adjusted-OR = 2.23; 95%CI = 1.01-4.92) and adenocarcinoma (adjusted-OR = 1.82; 95%CI = 0.90-3.66). Likewise, residents in urban or semi-urban areas displayed a non-significant increased risk of lung cancer (adjusted-OR = 1.33; 95%CI = 0.86-2.06 and adjusted-OR = 1.34; 95%CI = 0.86-2.07, respectively). In urban areas, however, there was a statistically significant risk of adenocarcinoma (adjusted-OR = 1.92; 95%CI = 1.09-3.38).

**Table 3:** Odds ratios of lung cancer, overall and by histologic subtype, in Asturias, by exposure category

	Total				Squamous cell carcinoma			Adenocarcinoma			Small cell carcinoma		
	**Controls**	**Cases**	**OR (95%CI)**^**a**^	**P-value**^**b**^	**Cases**	**OR (95%CI)**^**a**^	**P-value**^**b**^	**Cases**	**OR (95%CI)**^**a**^	**P-value**^**b**^	**Cases**	**OR (95%CI)**^**a**^	**P-value**^**b**^
	**N**	**N**			**N**			**N**			**N**		

**Unexposed**	437	416	Reference		171	Reference		114	Reference		74	Reference	
**Industrial**	63	74	1.49 (0.93-2.39)		20	0.98 (0.49-1.94)		20	1.82 (0.90-3.66)		18	2.23 (1.01-4.92)	
**Urban**	63	70	1.33 (0.86-2.06)		24	1.19 (0.65-2.21)		28	1.92 (1.09-3.38)		8	0.87 (0.36-2.10)	
**Semi-urban**	63	66	1.34 (0.86-2.07)	0.190	27	1.65 (0.92-2.97)	0.400	20	1.46 (0.78-2.72)	0.070	9	1.13 (0.50-2.58)	0.230

^a^ORs were estimated from a multiple logistic regression model that included age, sex, hospital area, tobacco consumption, family history of cancer, and occupation

^b^P-value for the whole effect of outdoor exposure

**Figure 2:**
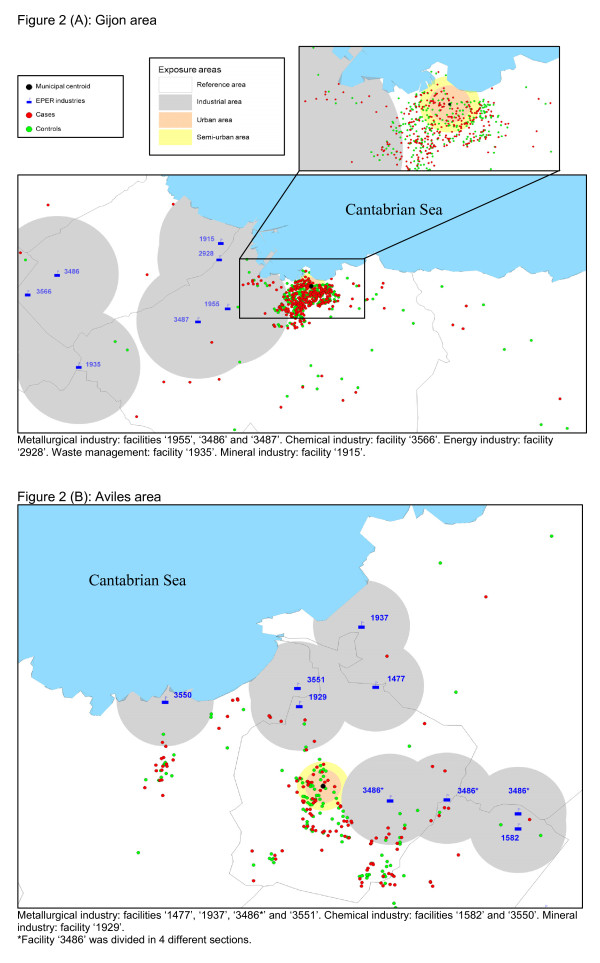
**Distribution of cases, controls, municipal centroids, and industries in the Gijon (A) and Aviles (B) health areas**. 2A: Metallurgical industry: facilities '1955', '3486' and '3487'. Chemical industry: facility '3566'. Energy industry: facility '2928'. Waste management: facility '1935'. Mineral industry: facility '1915'. 2B: Metallurgical industry: facilities '1477', '1937', '3486*' and '3551'. Chemical industry: facilities '1582' and '3550'. Mineral industry: facility '1929'. *Facility '3486' was divided in 4 different sections.

A separate analysis was conducted for the health catchment areas served by the Gijon and Aviles hospitals, areas that contributed more than 100 case-control pairs. Figures 2 (A) and 2(B) depict the distribution of cases, controls, municipal centroids and industries in the Gijon and Aviles health areas, respectively. Estimated ORs of lung cancer, both overall and by histologic subtype, are shown for these areas in Table 4. In the Gijon health area, individuals living in the urban area registered a statistically significant increased risk of lung cancer (adjusted-OR = 2.17; 95%CI = 1.25-3.76). ORs were high for all histologic subtypes, reaching statistical significance for the two most frequent subtypes, namely, squamous cell carcinoma (adjusted-OR = 3.07; 95%CI = 1.42-6.60) and adenocarcinoma (adjusted-OR = 2.01; 95%CI = 1.00-4.05).

**Table 4:** Odds ratios of lung cancer, overall and by histologic subtype, in Asturias' two main hospital catchment areas, by exposure category

	Total				Squamous cell carcinoma			Adenocarcinoma			Small cell carcinoma		
	**Controls**	**Cases**	**OR (95%CI)**^**a**^	**P-value**^**b**^	**Cases**	**OR (95%CI)**^**a**^	**P-value**^**b**^	**Cases**	**OR (95%CI)**^**a**^	**P-value**^**b**^	**Cases**	**OR (95%CI)**^**a**^	**P-value**^**b**^
	**N**	**N**			**N**			**N**			**N**		

**Gijon**													
**Unexposed**	248	211	Reference		80	Reference		75	Reference		36	Reference	
**Industrial**	36	41	1.51 (0.85-2.66)		15	1.57 (0.73-3.39)		13	1.01 (0.46-2.21)		10	2.17 (0.86-5.46)	
**Urban**	36	55	2.17 (1.25-3.76)		22	3.07 (1.42-6.60)		19	2.01 (1.00-4.05)		6	1.51 (0.52-4.37)	
**Semi-urban**	35	48	1.66 (0.94-2.92)	0.017	19	1.91 (0.90-4.03)	0.018	16	1.53 (0.72-3.22)	0.218	10	2.01 (0.81-5.03)	0.237

**Aviles**													
**Unexposed**	122	126	Reference		51	Reference		25	Reference		25	Reference	
**Industrial**	18	28	0.96 (0.45-2.06)		6	0.50 (0.16-1.58)		11	2.12 (0.80-5.65)		6	0.85 (0.25-2.86)	
**Urban**	18	13	0.95 (0.38-2.34)		6	0.88 (0.26-3.03)		6	1.70 (0.50-5.79)		0	-	
**Semi-urban**	18	9	0.56 (0.22-1.45)	0.693	1	0.19 (0.02-1.60)	0.214	3	1.05 (0.25-4.33)	0.453	2	0.63 (0.11-3.69)	0.238

^a^ORs were estimated from a multiple logistic regression model that included age, sex, tobacco consumption, family history of cancer, and occupation

^b^P-value for the whole effect of outdoor exposure

Residents living close to industrial facilities displayed a non-statistically significant excess risk of lung cancer (adjusted-OR = 1.51; 95%CI = 0.85-2.66). Finally, people living in semi-urban areas also showed a non-significant excess of risk of lung cancer (adjusted-OR = 1.66; 95%CI = 0.94-2.92).

In the Aviles health area, no statistically significant differences in risk were found for cases living in the different exposure categories studied. It is noteworthy that, though there was an excess of cases over controls in the industrial area (28 vs. 18), the adjusted OR was nevertheless slightly lower than unity (adjusted-OR = 0.96; 95%CI = 0.45-2.06). In the analysis by histologic type, residents in industrial and urban areas showed a non-statistically significant increased risk of adenocarcinoma (adjusted-OR = 2.12; 95%CI = 0.80-5.65 and adjusted-OR = 1.70; 95%CI = 0.50-4.33, respectively).

## Discussion

In this study, we investigated the effects of exposure to urban and industrial air pollution on lung cancer risk in an industrialized area of Northern Spain. Our findings support the hypothesis that air pollution might be a risk factor for lung cancer. Indeed, our analyses indicate excess of risk of lung cancer among residents in both urban and industrial areas, though estimates failed to attain statistical significance. However, separate analyses of the two main health areas targeted for study confirmed excess risk in the Gijon area, while results in Aviles were mainly negative.

Difficulties in assessing environmental exposure and its long-term effects have posed numerous methodological problems in epidemiologic studies, pertaining mainly to the use of aggregated data for exposure and the lack of information on relevant confounders. Pending the development of adequate biomarkers of exposure, some authors have argued that well designed case-control studies with improved methods for retrospective assessment of exposure to industrial pollution and potential confounding factors should be used [9,19]. To our knowledge, this is the first attempt to assess the influence of environmental pollution on lung cancer in Spain using individual data. The study design guarantees the availability of information on lung cancer risk factors, such as smoking habit and occupational exposure, which can be controlled for, and enables case and control exposures to be individually classified.

Insofar as environmental exposure is concerned, our measures were based on the residential location of the participants, and, despite the fact that were only able to take the geographical coordinates of subjects' last-reported residence into account, our study population proved to be very stable, i.e., 88.9% of cases and 89.3% of controls had lived in their last-reported residence for more than 5 years, and 80.0% of cases and 80.1% of controls had lived there for more than 10 years. We repeated the analyses with this last subgroup and we found similar results (data not shown). This variable affords relevant advantages for a case-control study, in that it cannot be expected to be influenced by recall bias. Moreover, the fact that we recruited incident cases also served to prevent possible changes of address associated with diagnosis of cancer. Hence, if there were any bias affecting proximity to pollution sources in relevant periods of life, our bias would be non-differential, causing an attenuation of the estimated effect.

Industrial pollution sources were identified using the EPER, which includes all industrial plants that have exceeded the reporting thresholds for one or more of the pollutants included in EU Decision 2000/479/EC. Subject to adequate validation of the geographical location of the data, this register has proved useful in ecological studies for ascertaining possible associations between residential proximity to such installations and mortality due to several cancers [17,36-39]. To our knowledge, ours is the first case-control study to use publicly-available EPER data to analyze the effects of industrial pollution on cancer, and lung cancer in particular. Although this register includes quantitative data on pollutant emissions, the fact that this information was reported voluntarily raises doubts about its reliability. As a result, we preferred to use distance to pollutant sources as a proxy of population exposure.

Inevitably, the use of hospital-based controls is a potential limitation. In our case, the hospitals where the cases were recruited were reference centers for all patients requiring hospitalization. Our controls were referred to these hospitals owing to the presence of acute health conditions thought to be unrelated to lung cancer risk factors. The geographic distribution of the control population likely reflects population density in the health areas studied. Although there is always a chance of recall bias being present, due to the fact that information on confounding variables was obtained retrospectively, the estimators obtained for the most important of these -tobacco exposure and occupation- were nevertheless in line with the literature.

Exposure to industrial pollution was defined by the distance to the nearest industrial facility. It would have been of interest to analyze subjects' proximity to every industrial facility but, when we tried to perform this type of analysis, two additional problems arose: first, few individuals lived in the vicinity of each industrial installation, thereby severely limiting the statistical power; and second, proximity among industries led the exposure areas of several facilities to overlap, thus rendering individual interpretation of results difficult. We decided to use the strategy proposed by Barbone, and proceeded to define areas of exposure based on the geographic distribution of our controls [24].

Exposure to urban air pollution has been associated with increased lung cancer risk. It is well established that urban and outdoor air contains known and suspected human carcinogens. Urban air contains benzo[α]pyrene, benzene, and 1,3-butadiene, together with carbon-based particles onto which carcinogens may be adsorbed, oxidants such as ozone and nitrogen dioxide, and sulfur and nitrogen oxides in particle form [40]. Outdoor air, particularly in densely populated urban environments, contains inorganic particulates (arsenic, asbestos, chromium and nickel), radionuclides (210Pb, 212Pb and 222Rn), and gaseous and particulate organic species (benzene, benzo[α]pyrene and benzene-soluble organics) [11]. These substances are present as components of complex mixtures proceeding basically from combustion of fossil fuels for power generation or transportation. In this respect, Gijon is the most heavily populated city in Asturias, with 262,470 inhabitants (24.3% of the region's total population) [28], and it ranks among the most polluted cities in Spain. SO2, NO2, and, in particular, PM10 levels, monitored since 2000, repeatedly exceed the range set for air quality standards by European Union Directive 99/30/EC [41].

Our findings for industrial pollution are consistent with previous studies, including two reviews [9,19], the former of which [19] reported in 1990 that most ecological studies showed an increased risk of lung cancer among populations living near non-ferrous smelters and a variety of other heavy industrial types. They noted, however, that few studies controlled for potential confounders, such as smoking or occupation. In 2001, Benedetti [9] reviewed 10 case-control studies: while seven of these reported an association between lung cancer risk and residential proximity to smelters, complex industrial areas, or localized sources of industrial emissions, three found little evidence of such an association. More recently, several studies have also observed associations between risk of lung cancer and: prolonged residence close to heavy industry [12]; residence within a 2-kilometer radius of a petrochemical plant in Brindisi (Italy) [13]; and residence in an urban area near a coke oven plant in Northern Italy [15].

Our results show that excess risks associated with urban and industrial pollution were concentrated in the Gijon health area, though in the case of industrial pollution the excess risk did not prove statistically significant. This area includes seven EPER industrial installations, four of which (two metal industries, a combustion installation, and a cement plant) could directly affect the general population due to their proximity to the city center. Several studies have reported excess lung cancer risks in the vicinity of these types of facilities. Metal industries located near the city center of Gijon include a steel foundry and an aluminum smelter, both associated with lung cancer risk in the literature. Indeed, steel founding is one of the industries classified by the International Agency for Research on Cancer (IARC) as implying a carcinogenic risk to humans [42], and several studies have reported an increased risk of lung cancer. These include a nested case-control study of Asturian iron and steel foundry workers [43] and a recent review of cohort studies conducted on workers exposed to PAHs [44]. Similarly, aluminum production was classified by the IARC as a group 1 carcinogen [42], and several studies have reported excess risk of lung cancer associated with exposure to substances released by aluminum smelters [45-47]. With respect to fossil fuel-fired electric power plants, it is well established that these emit known or suspected carcinogens [48], with several studies having observed high concentrations of heavy metals in areas exposed to pollution from coal-fired power installations [15,49], and a recent ecological study undertaken in Spain having reported excess lung cancer mortality among the population residing near Spanish combustion installations included in the EPER [17]. Finally, insofar as cement plants are concerned, Fano observed a significant excess risk of lung cancer among people living in the proximity of a cement plant [50], an ecological study conducted in Lithuania documented excess risk of lung cancer among male cement workers [51], and a recent IARC multicenter case-control study on occupation reported an elevated lung cancer risk among men involved in the cement industry [52]. Emissions from these industries include known or suspected carcinogens, such as arsenic, benzene, cadmium, chromium, dioxins, dichloromethane, lead, and nickel.

The Aviles health area contains nine EPER industrial installations (six metal industries, a glass installation, and two chemical plants), all of which are relatively far from the city center and, by extension, from the residence of most of the study population. Yet, the industries that are present in this area, mainly metal production and processing installations, have been associated with lung cancer risk in the literature. Nevertheless, the lack of association observed for the Aviles hospital area might also be due to lower statistical power, since we had only 176 cases and matched controls for this area.

Analyses by histologic type should be approached with care, owing to the small number of cases found in most categories of exposure. However, our results suggest that, while industrial air pollution may play a more specific role in the etiology of adenocarcinoma and small cell carcinoma, urban air pollution is associated with non-small cell lung cancer, whether squamous cell carcinoma or adenocarcinoma. The literature on histologic types of lung cancer and air pollution is limited. Barbone observed that air pollution was a moderate risk factor for certain histologic types of lung cancer in Trieste, Italy [24]. Urban air pollution appears to increase the risk of small cell and large cell carcinoma, while the effects of industrial air pollution vary with the industrial process. In this connection, Barbone et al., found excess risk of adenocarcinoma in the shipyard section and increased risk of all histologic types in the incinerator section. In an earlier study conducted in China, Xu et al., [53] found that the association with outdoor air pollution was stronger with squamous and oat cell cancer and adenocarcinoma. In occupational studies, Lubin and Blot [54] showed that both cigarette smoking and occupational exposure have stronger associations with squamous and small cell cancers than with adenocarcinoma. However, occupational exposure to asbestos appears to be more strongly associated with adenocarcinoma than with other types of lung cancer [55].

It is remarkable that the data on dispersion of industrial pollution emission may provide useful clues for evaluating results, but these are not available for the installations analyzed. Similarly, dispersion of the carcinogens present in air pollution is critically dependent on prevailing winds, with wind roses being one of the most useful tools for describing wind features. In Asturias, wind is a little-known climatic element owing to the small number of meteorological monitoring stations present in the region. The most remarkable and important fact is the pronounced seasonal nature of the wind, which makes it difficult to draw conclusions based on observation of prevailing winds in the area studied.

## Conclusions

In conclusion, our study furnishes further evidence that air pollution, both urban and industrial, is a moderate risk factor for lung cancer, which varies according to histologic type and health area. However, further research -particularly where it includes and makes use of the specific quantities of carcinogenic substances emitted- may be of value for assessing this relationship in greater depth.

## List of abbreviations

OR: odds ratio; 95%CI: 95% confidence interval; PAHs: polycyclic aromatic hydrocarbons; EPER: European Pollutant Emission Register; ICD-9: International Classification of Diseases-9th revision; PY: pack-years; SIGPAC: Sistema de Información Geográfica de Parcelas Agrícolas (Farm Plot Geographic Information System); UTM: Universal Transverse Mercator; IARC: International Agency for Research on Cancer

## Competing interests

The authors declare that they have no competing interests.

## Authors' contributions

MFLC, MP and AT conceived the idea and MFLC and JGP participated in the design of the analyses and draft the manuscript. JGP performed the statistical analysis. MP, BPG, NA, and GLA participated in the design of the analyses and revised the manuscript. All authors read and approved the final manuscript.
